# Genetic Structure of Pacific Trout at the Extreme Southern End of Their Native Range

**DOI:** 10.1371/journal.pone.0141775

**Published:** 2015-10-28

**Authors:** Alicia Abadía-Cardoso, John Carlos Garza, Richard L. Mayden, Francisco Javier García de León

**Affiliations:** 1 Fisheries Ecology Division, Southwest Fisheries Science Center, National Marine Fisheries Service, Santa Cruz, California, United States of America; 2 Institute of Marine Sciences, University of California Santa Cruz, Santa Cruz, California, United States of America; 3 Department of Biology, Saint Louis University, St. Louis, Missouri, United States of America; 4 Laboratorio de Genética para la Conservación, Centro de Investigaciones Biológicas del Noroeste, S.C., La Paz, Baja California Sur, Mexico; Swansea University, UNITED KINGDOM

## Abstract

Salmonid fishes are cold water piscivores with a native distribution spanning nearly the entire temperate and subarctic northern hemisphere. Trout in the genus *Oncorhynchus* are the most widespread salmonid fishes and are among the most important fish species in the world, due to their extensive use in aquaculture and valuable fisheries. Trout that inhabit northwestern Mexico are the southernmost native salmonid populations in the world, and the least studied in North America. They are unfortunately also facing threats to their continued existence. Previous work has described one endemic species, the Mexican golden trout (*O*. *chrysogaster*), and one endemic subspecies, Nelson’s trout (*O*. *mykiss nelsoni*), in Mexico, but previous work indicated that there is vastly more biodiversity in this group than formally described. Here we conducted a comprehensive genetic analysis of this important group of fishes using novel genetic markers and techniques to elucidate the biodiversity of trout inhabiting northwestern Mexico, examine genetic population structure of Mexican trout and their relationships to other species of Pacific trout, and measure introgression from non-native hatchery rainbow trout. We confirmed substantial genetic diversity and extremely strong genetic differentiation present in the Mexican trout complex, not only between basins but also between some locations within basins, with at least four species-level taxa present. We also revealed significant divergence between Mexican trout and other trout species and found that introgression from non-native rainbow trout is present but limited, and that the genetic integrity of native trout is still maintained in most locations. This information will help to guide effective conservation strategies for this important group of fishes.

## Introduction

**Fig 1:**
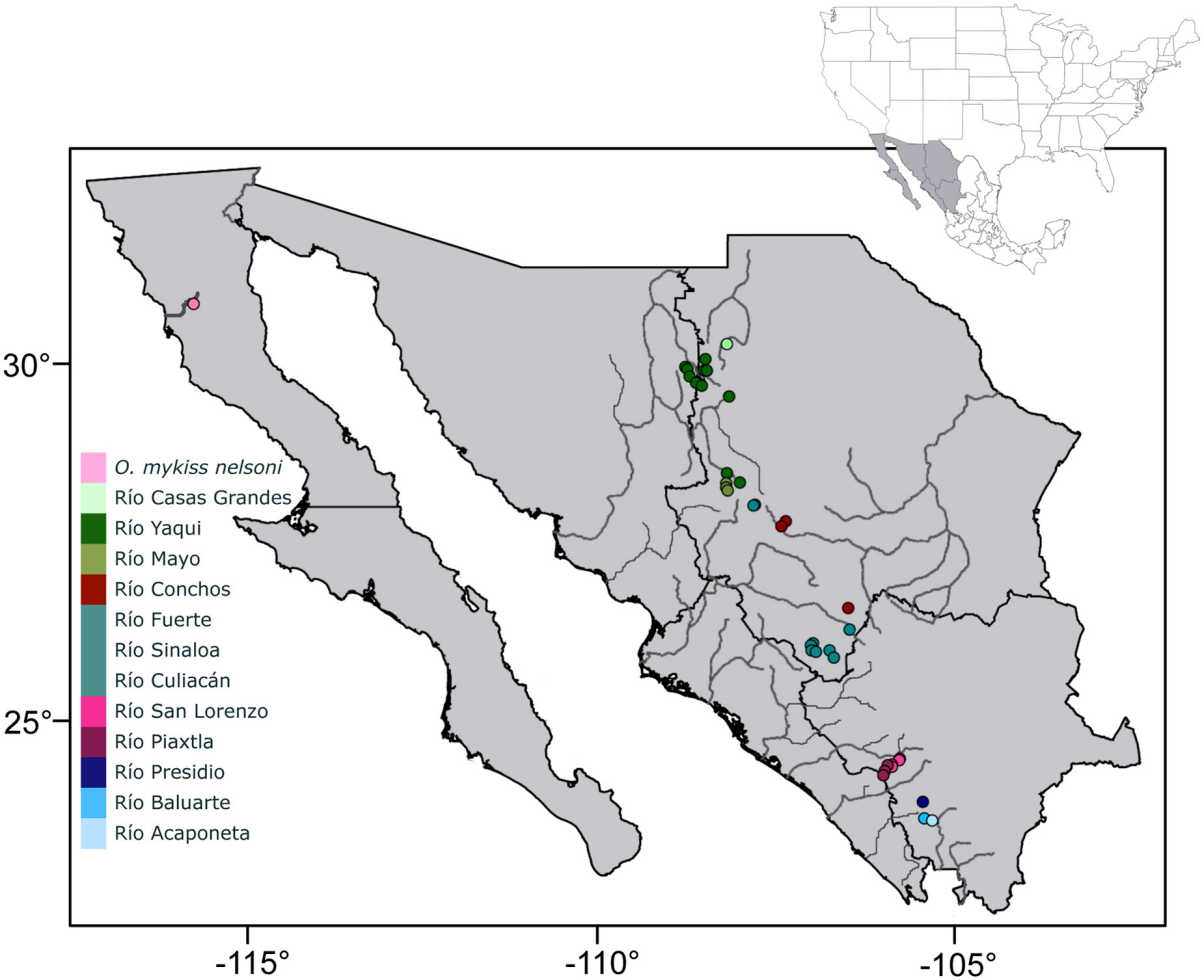
Geographic locations of sampling sites from 13 major drainages in northwestern Mexico.

The first step in construction of an effective conservation strategy is to document the diversity of biological units in the focal taxa and gain an understanding of the evolutionary relationships among them and their close relatives [1]. The taxonomic status of trout inhabiting northwestern Mexico has been the subject of speculation and controversy for decades and Pacific trout expert Behnke [2] considered this group as “the most diverse and the least known trout of western North America”. Only two trout taxa from Mexico have been formally described: Nelson’s trout, *Oncorhynchus mykiss nelsoni* [3], distributed in the Río Santo Domingo in northern Baja California (Fig 1); and the Mexican golden trout, *O*. *chrysogaster* [4] from the ríos Fuerte, Sinaloa and Culiacán in the central highlands of the Sierra Madre Occidental (SMO). Both taxa are currently protected by Mexican law [5] and the Mexican golden trout was listed as Vulnerable by the IUCN in 1990 [6]. Other groups of trout are found throughout the SMO, including in three basins north of the range of the Mexican golden trout (NSMO here after) and in five basins to the south (SSMO here after). Two populations of trout have also been discovered in the Río Conchos, which unlike all the other trout basins in Mexico, drains into the Atlantic Ocean. The NSMO drainages include ríos Casas Grandes, Mayo and Yaqui. The SSMO basins include ríos San Lorenzo, Piaxtla, Presidio, Baluarte, and Acaponeta [2,7–9] and trout may also occur farther to the south in the Río San Pedro Mezquital (Espinoza-Pérez, pers. comm.). These trout have been considered as undescribed subspecies of *O*. *mykiss*, but no formal taxonomic evaluation has been conducted and their taxonomic status is not clear [2,10].

The fossil record indicates that trout inhabited Mexico during the Pleistocene and the southern-most record for a fish assigned to the family Salmonidae is ~ 20° North latitude, in the Lake Chapala, Jalisco region [11]. Behnke [12] suggested that the Gulf of California acted as a refugium for anadromous trout during the Pleistocene glaciations and that these trout migrated from the Gulf into streams that drain what is now northwestern Mexico, Arizona and New Mexico. Subsequent increases in both ocean and river water temperatures constrained these trout to high elevation headwaters of different river systems and the potentially long periods of isolation resulted in the evolution of the Gila (*O*. *gilae*), Apache (*O*. *apache*), Mexican Golden (*O*. *chrysogaster*) trout and, presumably, the other groups of trout in the SMO.

Only one genetic study, using mitochondrial DNA (mtDNA) [13], has examined phylogenetic relationships among Mexican trout, and a few others have examined relationships of one or two populations of trout from Mexico to other trout in North America. These latter studies utilized data from karyology [14], allozymes [15], mtDNA [16] and microsatellites [10,17,18], but most of them focused on just one group (*i*.*e*., *O*. *chrysogaster*) or a few populations in specific regions (*i*.*e*., trout from the ríos Yaqui or Mayo). While these studies provided valuable information and some insight into the diversity of trout inhabiting northwestern Mexico, incomplete sampling, small sample sizes, and the low resolution of some of these analyses have left many unresolved questions. Thus, there is a clear need for more information on genetic diversity, population structure and evolutionary relationships (sensu [1]) of trout in Mexico, as they are the southernmost populations of salmonid fish in their native range (the north Pacific Ocean and associated tributaries), and are the only fish in this group that inhabit subtropical waters. Given the importance of trout in global aquaculture and fisheries [19], and impending climate change, it is critical to understand adaptation to such conditions in these primarily cold-water fishes.

As in many parts of the world, anthropogenic factors including habitat loss, logging, pollution and global climate change threaten Mexican freshwater ecosystems, and without pressing conservation actions for some populations, the unique gene pool represented by these sub-tropical trout may be lost. Conservation of the trout inhabiting the river basins of northwestern Mexico requires documentation of the genetic diversity of this species complex, as well as an understanding of the evolutionary relationships of this group. Such a comprehensive analysis requires sampling of fish from all river basins in Mexico where these trout have been documented and comparisons with other Pacific trout species: *O*. *mykiss*, *O*. *clarkii*, *O*. *gilae* and *O*. *apache*.

In addition, the practice of introducing exotic hatchery rainbow trout (*O*. *mykiss irideus*) has led to establishment of naturalized populations in several drainages where native Mexican trout also occur [7,9,18]. Fish produced at hatcheries face problems such as domestication selection, inbreeding depression, reduced genetic variation, and increased susceptibility to disease. Introductions of non-native species or strains can have devastating effects on native species, particularly when they are closely related. These effects can range from reduction of native genetic diversity to extinction through hybridization of local populations [20,21]. Introgression from genetically depauperate hatchery rainbow trout into native trout populations in California has been reported [22,23], and this may also be a substantial threat to some native Mexican trout. In addition, hybridization of introduced rainbow trout with native westslope cutthroat trout (*O*. *clarkii lewisi*) has been ubiquitous [24–27], with many populations at risk of extinction from this hybridization [28]. Native *O*. *apache* and *O*. *gilae* trout populations are similarly affected, with both species receiving protection under the United States Endangered Species Act (ESA) due to habitat loss, hybridization and genetic introgression with introduced rainbow trout [29]. Approximately 65% of *O*. *apache* populations have some degree of introgression [20], and at least two populations of *O*. *gilae* have been lost due to hybridization [21]. A recent genetic study found some evidence of introgression of Mexican trout populations by hatchery rainbow trout but it was generally restricted to the areas around the hatcheries [30]. However, it is critical to further examine the existence and extent of introgression of hatchery rainbow trout from a broad range of potential source stocks into populations of Mexican native trout to fully understand the extent of this threat and take appropriate conservation actions.

Here, we pursue three primary goals: 1) to document the genetic biodiversity of native trout in northwestern Mexico, 2) evaluate relationships between these trout populations and their potential evolutionary origins, and 3) evaluate the extent of hybridization and genetic introgression from hatchery rainbow trout into native trout in northwestern Mexico. To investigate these questions, we use data from a large number of genetic markers, including both microsatellites and single nucleotide polymorphisms, and analyze samples from all the basins in Mexico in which native trout have been recently documented.

## Materials and Methods

### Tissue collection and DNA extraction

Permits for the collections were issued by SEMARNAT (Dirección General de Vida Silvestre, Secretaría de Medio Ambiente y Recursos Naturales, License No. FAUT 0117 Responsible: Héctor Espinosa Pérez) and Permisos de Pesca de Fomento DGOPA 21205/0765, 250406/1606 030506/1677, 240800 213-03/2366, 300801-613-03/1607. The study protocols (#1427, 1595, 1626) were approved by the Saint Louis University Animal Care and Use Committee.

Exhaustive sampling efforts between 1994 and 2010 by the binational group “Truchas Mexicanas” resulted in collection of a total of 914 tissue samples (≈1cm^2^) from trout at 42 localities (Fig 1; 13 basins represented). These streams are primarily in the Sierra Madre Occidental mountain range in the Mexican states of Chihuahua, Durango, Sinaloa and Nayarit, and in the Sierra de San Pedro Mártir mountain range, in the state of Baja California (S1 Table; Fig 1). Extremely rugged terrain and large cliffs characterize these regions and the stream localities were at elevations between 1,500 and 2,400 meters above sea level. The annual mean air temperatures at this altitude range from 3 to 20°C but below freezing temperatures can occur during wintertime.

Also, 147 tissue samples from hatchery rainbow trout were obtained from four hatcheries located in different basins in Mexico where native trout have been reported, and one hatchery located in Guachochi, Chihuahua which is oriented towards native trout conservation (S1 Table; see [7,18,30] for details on sample collection methods). Also, tissue samples were obtained from five *O*. *apache* (West Fork Black River population) and five *O*. *gilae* individuals (Gila River population).

Additionally, genotype data from 18 natural origin *O*. *mykiss* populations (*N* = 675) from California, USA that represent six Distinct Population Segments (DPSs) [31], and four *O*. *mykiss* hatchery strains (*N* = 187) were included in the analyses. Genotype data from five cutthroat trout subspecies—coastal: *O*. *c*. *clarkii*; (*N* = 47), Yellowstone: *O*. *clarkii bouvieri* (*N* = 20), Bonneville: *O*. *clarkii utah* (*N* = 16), Rio Grande: *O*. *clarkii virginalis* (*N* = 10), and Colorado: *O*. *clarkii pleuriticus* (*N* = 8)—were also incorporated (S1 Table). All populations were selected to be representative of their respective taxa, as determined by previous studies [23,32–35]. Genomic DNA was extracted from all samples using either a standard phenol/chloroform protocol or with a semi-automated membrane-based system (DNeasy 96 Tissue Kit) on a BioRobot 3000 (Qiagen Inc.).

### Microsatellite and single nucleotide polymorphism genotyping

All individuals were genotyped at 18 microsatellite loci identified in salmonid species: Omy27 [36], Ssa289 [37]; Omy77 [38]; Ssa85 [39]; One11b, One13b [40]; Omy1011 [41]; Ots103 [42]; Oki23 [43]; Ots1b [44]; OtsG3, OtsG43, OtsG85, OtsG243, OtsG249b, OtsG253, OtsG401, OtsG409 [45]. This set of loci has proven to be highly informative in the study of *O*. *mykiss* population structure and interactions among different groups of trout in California, including for the detection of introgression by all major lineages of hatchery rainbow trout [23,34,35,46], and some of these markers are also informative in *O*. *clarkii* [47,48].

PCR was conducted in 15μL volumes with 6.9μL H_2_O, 1.5μL 10X PCR buffer (Life Technologies Inc.), 0.9μM MgCl_2_, 0.6μM dNTPs, 1μM fluorescently labeled oligonucleotide primers, 0.04 U Amplitaq DNA polymerase (Life Technologies) and 4μL template DNA of variable concentration. Thermal cycling conditions are available from the authors upon request. PCR products were electrophoresed on an ABI377 or an ABI3730 Genetic Analyzer. Allele sizes were determined with Genotyper or Genemapper software (Applied Biosystems Inc.) and confirmed by two people independently.

A total of 93 single nucleotide polymorphism (SNP) loci were also genotyped on all samples. These SNPs include loci described by Aguilar and Garza [49], Campbell *et al*. [50], and Abadía-Cardoso *et al*. [51]. They have been validated in many populations of *O*. *mykiss* from California, Oregon and Washington [51], as well as in introduced populations in other parts of the world (unpublished data). A PCR pre-amplification was carried out in 5.4μL volume containing 2.5μL of 2X Master Mix (Qiagen), 1.3μL of pooled oligonucleotide primers, and 1.6μL template DNA. Pre-amplification thermal cycling conditions included an initial denaturation of 15 min at 95°C, and 13 cycles of 15s at 95°C, 4 min at 60°C (+ 1°C/cycle). Pre-amplification products were diluted 1:3 in 2mM Tris. Genotyping used 5’ nuclease allelic discrimination (TaqMan^™^) assays (Life Technologies) and was carried out in 96.96 Dynamic SNP Genotyping Arrays on an EP1 System (Fluidigm Corporation) using the manufacturer’s recommended protocols.

### Data analysis

The two classes of genetic markers used have important differences, such as higher polymorphism for microsatellites and a lower mutation rate for SNPs, among others. Such differences could provide distinct and complementary information about the genetic relationships and history of these trout. Therefore, some analyses were performed using only the SNP or microsatellite data. All analyses included all populations, regardless of sample size, except where indicated.

Within population genetic variation was examined using different estimators. Expected (*H*_*E*_) and observed (*H*_*O*_) heterozygosities [52] were estimated for microsatellites and SNPs separately using GENEPOP [53]. Percentage of SNP loci polymorphic at frequencies of 0.95 *P*(0.95) and 0.99 *P*(0.99) were calculated using GENETIX 4.05 [54]. The R-based package hierfstat [55,56] was used to estimate microsatellite allelic richness (*A*_*R*_) by rarefaction to account for sample size differences. A Bayesian model-based clustering analysis of individual ancestry implemented in the program STRUCTURE 2.2 [57] was also performed. This analysis was based on individual multilocus genotypes without using geographic information of the populations of origin for individual fish. Values of *K* = 2–7 were evaluated, with 20 iterations for each value and a burn-in period of 50,000 steps and 150,000 Markov Chain Monte Carlo replicates. The results were reordered and visualized using the software CLUMPP [58] and DISTRUCT [59].

Genetic relationships among populations were examined using three methods: a) Pairwise *F*_*ST*_ estimates and their significance, evaluated with 10,000 permutations using Arlequin 3.5 [60]; b) Principal components analysis (PCA) using the R-based package adegenet 1.3–4 [61], which identifies and summarizes joint relationships of the markers and differences between individuals [62]; and c) Unrooted neighbor-joining dendrograms using the combined dataset and the microsatellite data only, created using PHYLIP [63]. Markers that failed for any entire population were excluded from this last analysis, leaving 12 microsatellite and 85 SNP loci. Gila, Apache and Colorado River Cutthroat trout populations were excluded because of small sample sizes. The Cavalli-Sforza and Edwards [64] method was used to estimate pairwise genetic distances and 1,000 bootstrapped distance matrices used to evaluate node support.

Due to evident establishment of non-native hatchery rainbow trout in some of the main drainages of northwestern Mexico [30], genetic introgression from hatchery trout into naturally spawning Mexican trout populations was estimated using two different approaches: an ancestry analysis with STRUCTURE (*K* = 2–5, five iterations each) and PCA. These analyses were performed using all the naturally spawning populations from a basin (*e*.*g*. Río San Lorenzo) and data from the fish from hatcheries in that basin (*e*.*g*. “Piscicultura Vencedores” hatchery) as well as from the hatchery trout strains from the United States. The q-value from this clustering analysis with *K* = 2 was used as a measure of introgression. Even though samples from hatcheries located in the southernmost basins were not available, introgression from hatchery rainbow trout in the ríos Presidio, Baluarte, and Acaponeta was evaluated based on the results obtained in the analyses described above.

## Results

A total of 1,999 trout were successfully genotyped with the microsatellite panel, and 1,985 with the SNP panel. Those individuals that had excessive missing data (≥ 10 missing SNP loci or ≥ 9 missing microsatellite loci) were excluded from analyses (S1 Table).

**Fig 2:**
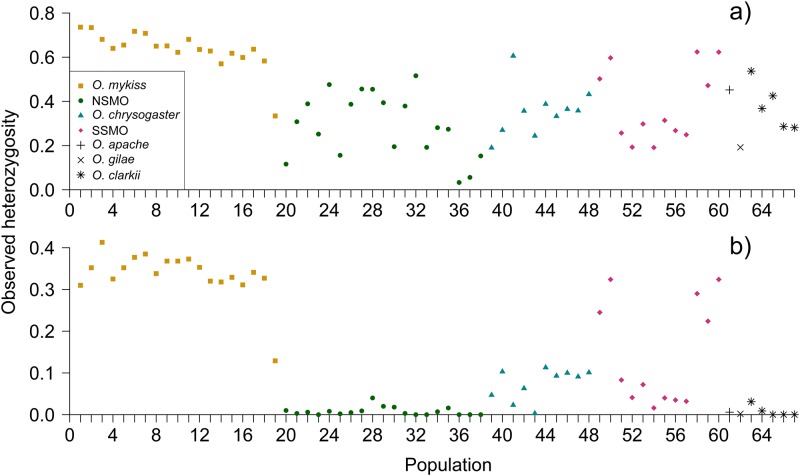
Mean observed heterozygosity (*H*_*O*_) per population in trout from northwestern Mexico and California, USA. a) Microsatellite data; b) SNP data. Populations are organized from north to south except for populations 61 to 67, which correspond to *O*. *apache*, *O*. *gilae*, and *O*. *clarkii*. NSMO: Northern Sierra Madre Occidental; SSMO: Southern Sierra Madre Occidental.

Observed heterozygosity per population with microsatellites ranged from 0.033 in Río Conchos-Arroyo Ureyna to 0.736 in Klamath River-Blue Creek, and with SNPs ranged from zero in several NSMO populations and *O*. *clarkii* ssp. to 0.413 in Gualala River-Fuller Creek (S2 Table; Fig 2). Overall, heterozygosity was higher for both marker types in *O*. *mykiss* populations (microsatellites: mean *H*_*O*_ = 0.636, range = 0.334–0.736; SNPs: mean *H*_*O*_ = 0.336, range = 0.129–0.413) than in any of the SMO populations (microsatellites: NSMO mean *H*_*O*_ = 0.288, range = 0.033–0.516; *O*. *chrysogaster* mean *H*_*O*_ = 0.354, range = 0.190–0.606; SSMO mean *H*_*O*_ = 0.382, range = 0.191–0.624; SNPs: NSMO mean *H*_*O*_ = 0.008, range = 0–0.040; *O*. *chrysogaster* mean *H*_*O*_ = 0.074, range = 0.003–0.113; SSMO mean *H*_*O*_ = 0.144, range = 0.016–0.324; Fig 2), and than in the other formally described species (microsatellites: *O*. *apache H*_*O*_ = 0.440; *O*. *gilae H*_*O*_ = 0.192; *O*. *clarkii* ssp. mean *H*_*O*_ = 0.379, range = 0.281–0.537; SNPs: *O*. *apache H*_*O*_ = 0.006; *O*. *gilae H*_*O*_ = 0.002; *O*. *clarkii* ssp. mean *H*_*O*_ = 0.008, range = 0–0.031; S2 Table; Fig 2).

**Fig 3:**
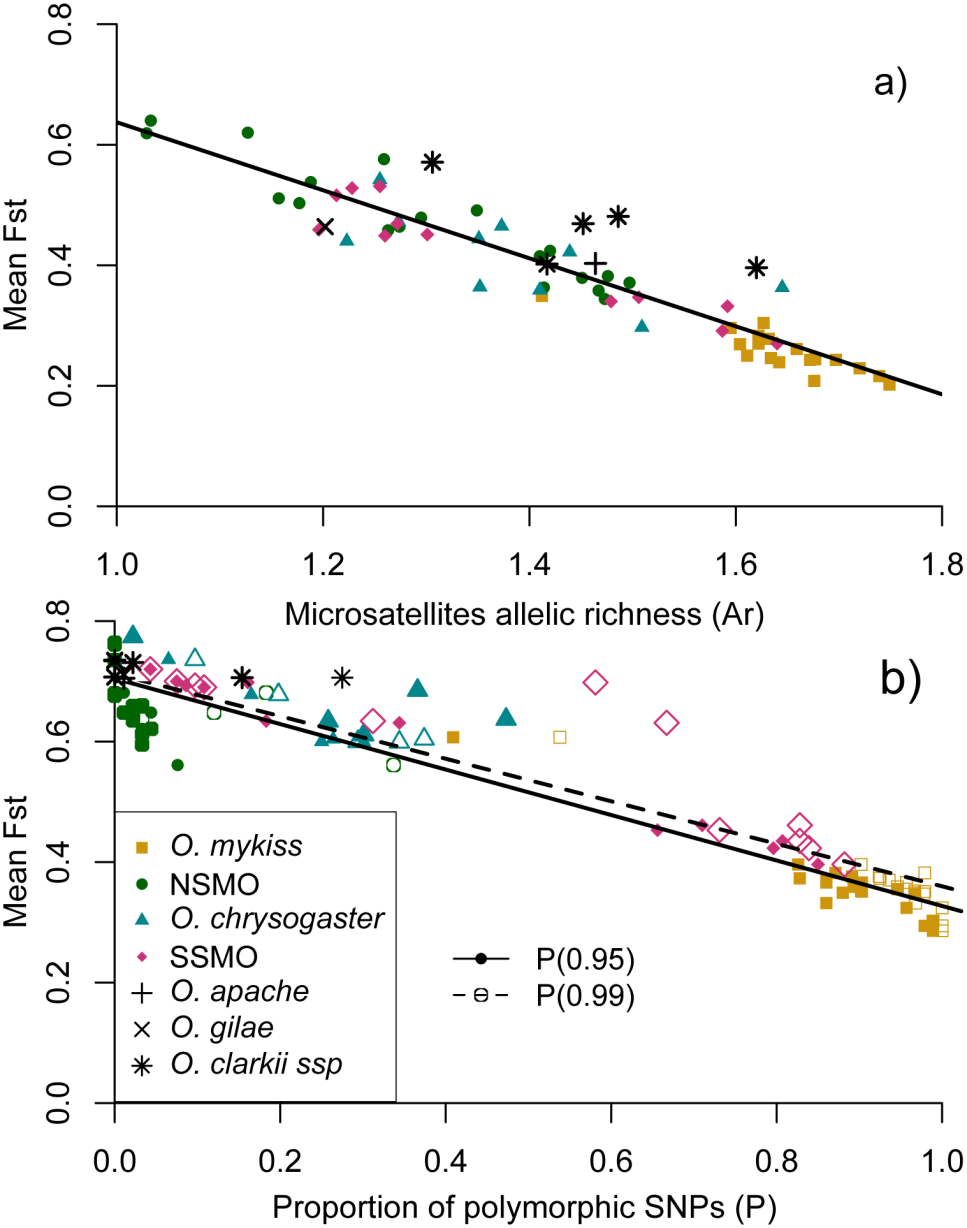
Correlation between mean *F*_*ST*_ and genetic diversity. a) Microsatellite allelic richness and b) percentage of SNPs polymorphic at frequencies of 0.95 and 0.99. Populations are organized from north to south except for populations 61 to 67, which correspond to *O*. *apache*, *O*. *gilae*, and *O*. *clarkii*. NSMO: Northern Sierra Madre Occidental; SSMO: Southern Sierra Madre Occidental.

Mean number of alleles per microsatellite locus and *A*_*R*_ were highest in *O*. *mykiss* populations (mean alleles/locus = 7.74; mean *A*_*R*_ = 1.65) and lowest in the Río Conchos-Río Rituchi population (alleles/locus = 1.06; *A*_*R*_ = 1.03; S2 Table; Fig 3). Within Mexican trout, the highest number of alleles per locus and highest *A*_*R*_ were observed in Río Fuerte-Río Verde (alleles/locus = 8.0; *A*_*R*_ = 1.65; S2 Table).

Percentage of polymorphic SNP loci at *P*(0.95) ranged from 0 to 0.99 and at *P*(0.99) from 0 to 1, with the highest values again observed in *O*. *mykiss* (mean *P*(0.95) = 0.88; mean *P*(0.99) = 0.95) and the lowest in NSMO fish (mean *P*(0.95) = 0.02; mean *P*(0.99) = 0.05), where all loci were monomorphic in several populations (S2 Table; Fig 3).

We observed higher mean pairwise population *F*_*ST*_ values with SNPs than microsatellites (S2 Table) and significant negative correlations were found when *F*_*ST*_ values were compared to both *A*_*R*_ (F_1,74_ = 517.8, R^2^ = 0.875, *p <* 0.001) and percentage of polymorphic SNPs (*P*(0.95): F_1,74_ = 859.5, R^2^ = 0.921, *p <* 0.001; *P*(0.99): F_1,74_ = 591.6, R^2^ = 0.889, *p <* 0.001; Fig 3). This was not unexpected, since the level of heterozygosity directly affects measures of differentiation among groups [65].

**Fig 4:**
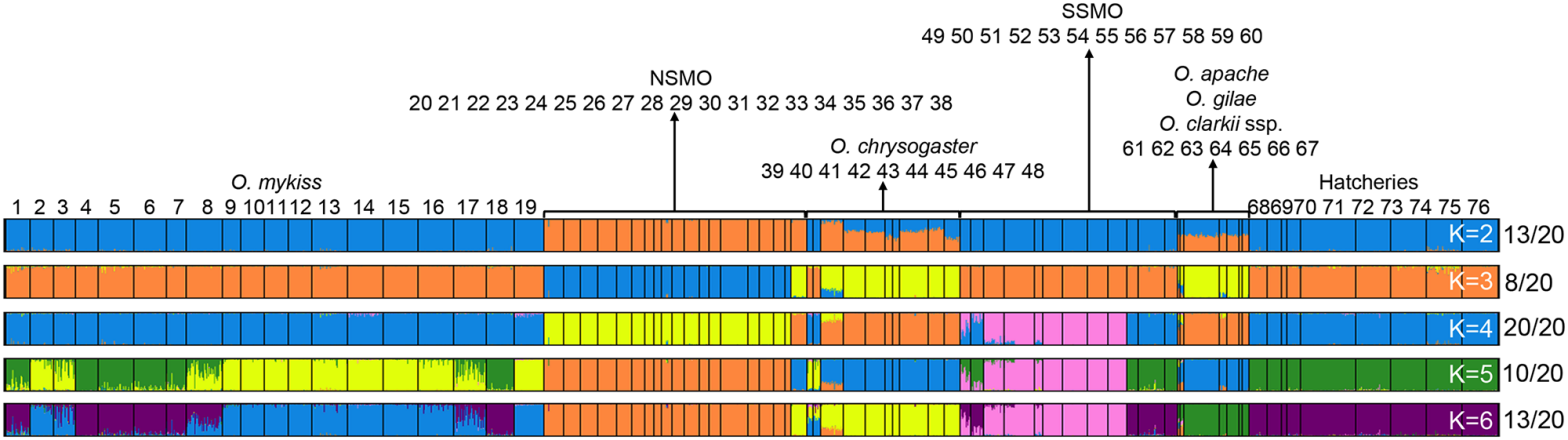
STRUCTURE analysis. Estimated membership fraction (represented by color proportions) of 1,932 individual fish from 18 *O*. *mykiss* populations, 42 trout populations from northwestern Mexico, one *O*. *gilae* and one *O*. *apache* population, five *O*. *clarkii* subspecies, and five Mexican and four U.S. hatchery rainbow trout stocks, using data from 18 microsatellites and 93 SNPs. Horizontal plots represent individual ancestry estimates. Each thin, colored, vertical line represents one individual. Vertical black lines separate collection localities. A summary of the 20 runs for each *K* value (*K* = 2–7) is shown. The right column indicates the number of observations for that general pattern. NSMO: Northern Sierra Madre Occidental; SSMO: Southern Sierra Madre Occidental. Numbers on top represent the “Population number” in S1 Table.

The STRUCTURE analysis clustered nearly all individuals according to their geographic location of capture. However, not all populations clustered by nominal species, as *O*. *clarkii* ssp. appeared to share some recent ancestry with *O*. *chrysogaster*, while *O*. *gilae* and *O*. *apache* shared some recent ancestry with *O*. *mykiss*, *O*. *chrysogaster*, and NSMO trout (Fig 4). Some general patterns could be observed across the different *K* values. For example, clear breaks occurred between *O*. *mykiss*, NSMO, *O*. *chrysogaster*, and populations from the ríos San Lorenzo/Piaxtla. Within the Río Conchos populations, a complicated pattern was observed: ríos Rituchi and Ureyna clustered with the rest of the NSMO complex but, in contrast, Arroyo El Molino shared recent ancestry with both the NSMO complex and *O*. *chrysogaster*, as did the population from Río Fuerte-Río Verde, which is nominally *O*. *chrysogaster*. Within *O*. *chrysogaster*, there were two other populations with anomalous patterns; Río Fuerte-Arroyo Aparique and Río Fuerte-Arroyo San Vicente clearly shared recent ancestry with *O*. *mykiss* -possibly an indication of genetic introgression. Populations from the ríos Presidio, Baluarte and Acaponeta also showed recent shared ancestry with *O*. *mykiss* and more specifically with the Central Valley DPS populations and hatchery strains (Fig 4).

Highly significant genetic differentiation between populations was also documented with the pairwise *F*_*ST*_ values (S3 Table), with the largest values observed among SMO populations (mean pairwise *F*_*ST*_ = 0.39–0.73) and between SMO and *O*. *mykiss* populations.

**Fig 5:**
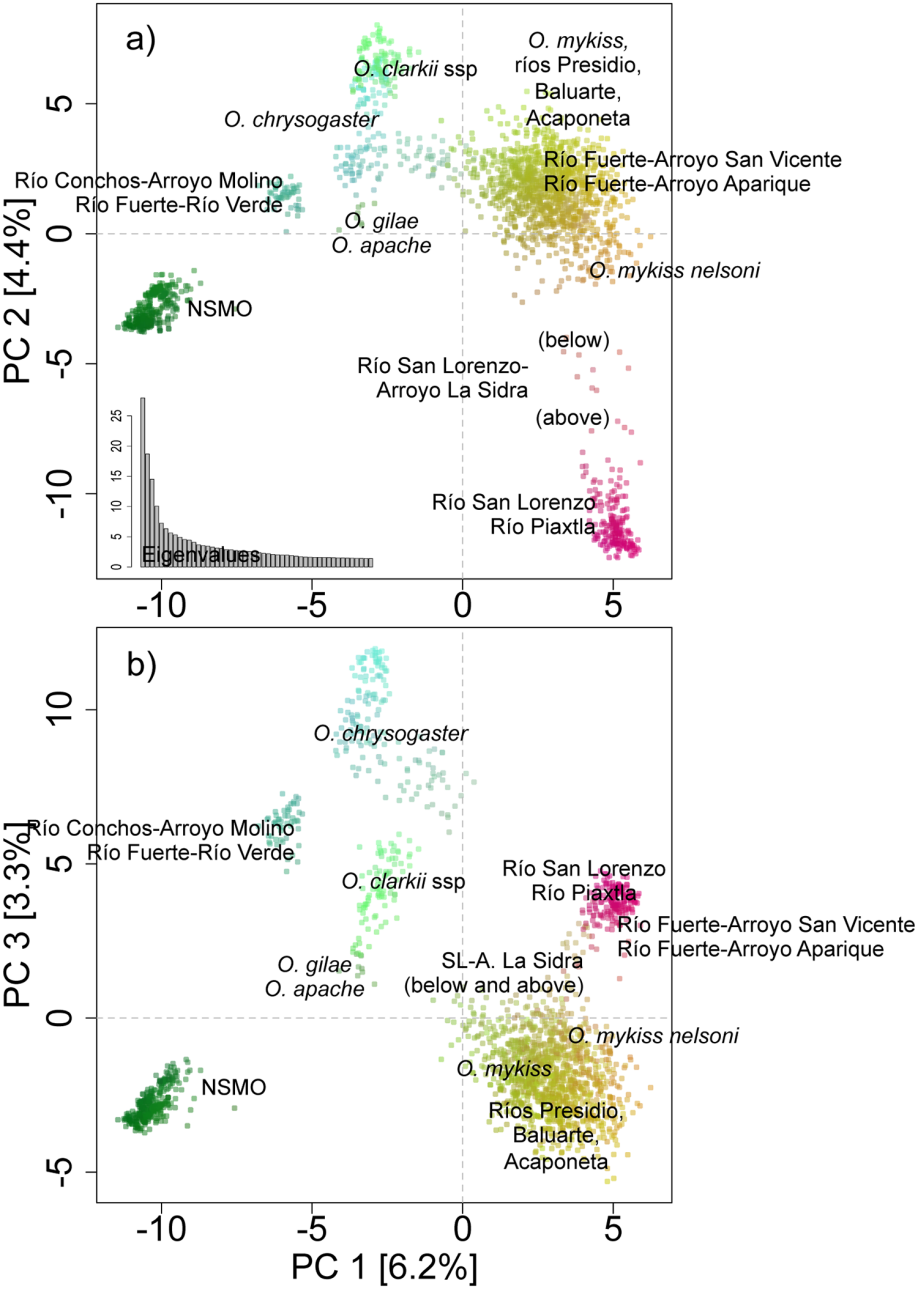
Principal components analysis of allele frequencies from 18 microsatellites and 93 SNPs and the first 50 eigenvalues. a) First (PC1) and second (PC2) principal components and b) first (PC1) and third (PC3) principal components. The difference in color (red, blue and green) between clusters indicates divergence using the first three principal components. Seven clusters are shown (see text for description of cluster membership). NSMO: Northern Sierra Madre Occidental.

The PCA revealed a number of well-differentiated clusters (Fig 5). One cluster (dark green) corresponds to the NSMO, and comprised all the populations from Río Yaqui tributaries (ríos Bavispe and Sirupa), Río Casas Grandes, Río Mayo, and two tributaries from the Río Conchos (ríos Rituchi and Ureyna). Another cluster (dark pink) included populations from all tributaries of the ríos Piaxtla and San Lorenzo except for Arroyo La Sidra (above and below waterfall; see below for more information about this locality). A third cluster (yellow/orange) encompassed all *O*. *mykiss* populations, including *O*. *m*. *nelsoni*, all hatchery stocks, as well as populations from the southernmost localities (ríos Presidio, Baluarte and Acaponeta). A fourth cluster (green/blue) included populations from two localities: Río Conchos-Arroyo El Molino and *O*. *chrysogaster* from the Río Fuerte-Río Verde. The remaining populations of *O*. *chrysogaster* formed a fifth group (light blue), except for populations from two tributaries of the Río Fuerte (arroyos Aparique and San Vicente), which clustered with the *O*. *mykiss* group with PC1 and PC2 (Fig 5a) and with the San Lorenzo/Piaxtla group with PC1 and PC3 (Fig 5b). Finally, *O*. *gilae* and *O*. *apache* trout defined a sixth group (olive green) and the *O*. *clarkii* subspecies (light green) overlapped with two *O*. *chrysogaster* populations (Río Sinaloa-Arroyo El Potrero and Río Fuerte-Arroyo Las Truchas) with PC1 and PC2 (Fig 5a) but were separated with PC1 and PC3 (Fig 5b).

**Fig 6:**
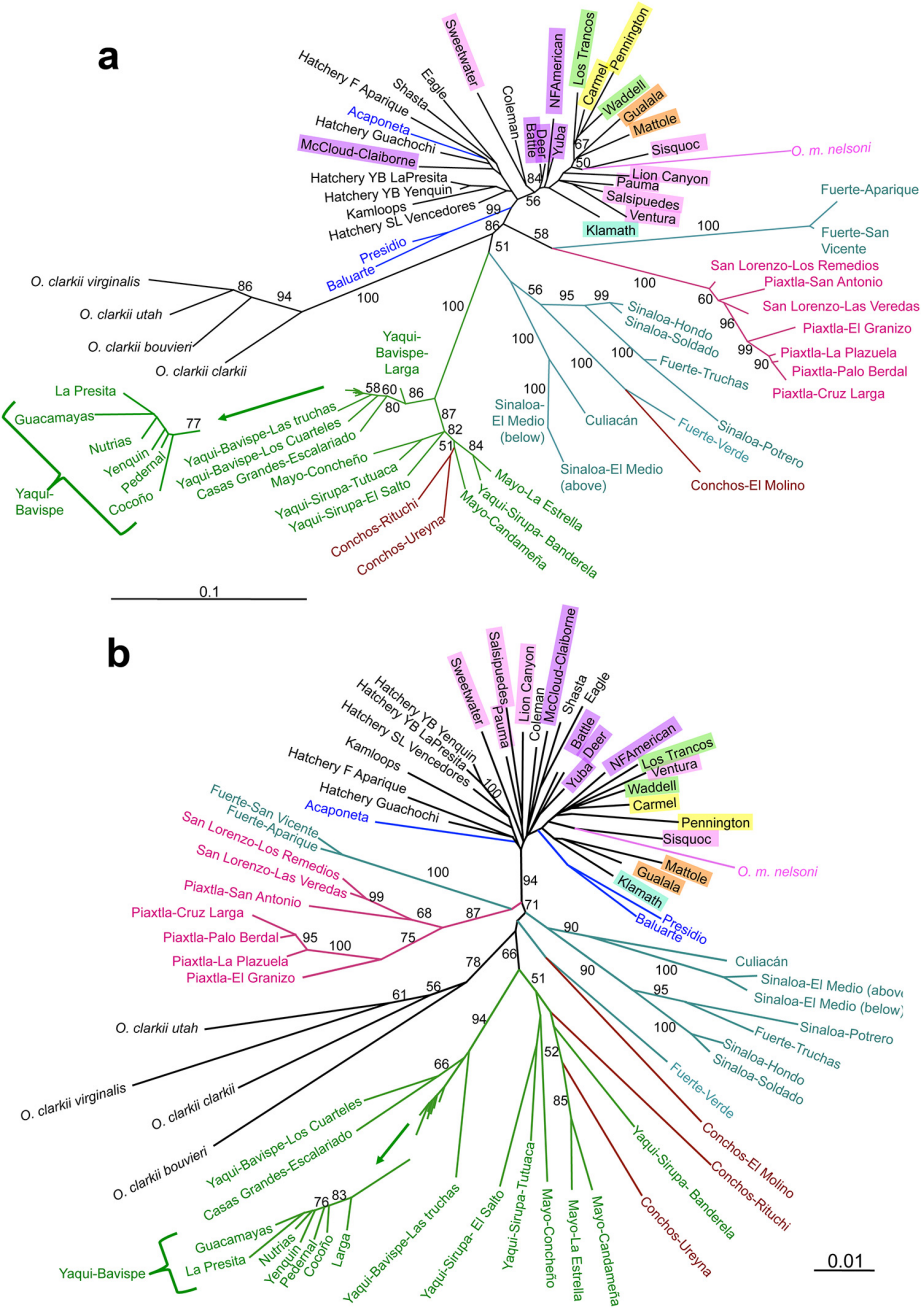
Unrooted neighbor-joining dendrograms. a) using data from both 12 microsatellites and 85 SNPs combined and b) using data from 12 microsatellite markers. The dendrogram was constructed with pairwise chord distances and bootstrap values are from 1,000 distance matrices constructed from bootstrap samples of the data from 18 *O*. *mykiss*, 19 NSMO, 10 *O*. *chrysogaster*, and 12 SSMO natural-origin populations, four *O*. *clarkii* subspecies, and five Mexican and four U.S. hatchery stocks. Bootstrap support > 50% percent is indicated for Mexican trout groupings (for *O*. *mykiss* see Garza *et al*. 2014). DPS affiliations of California *O*. *mykiss* populations (creeks) are highlighted in colors. Mexican natural-origin populations are indicated with branches and names colored.

Topologies of the two unrooted dendrograms (Fig 6) were generally concordant, with the exception of the populations from the southern ríos Presidio and Baluarte that clustered within the *O*. *mykiss* lineage on the combined tree (Fig 6a), but formed a separate group (but with low bootstrap support) on the microsatellite tree (Fig 6b).

Several notable features could be identified in the population groupings of both dendrograms (Fig 6). First, the topology observed was mostly consistent with the designation of populations into the different named species and was also consistent with the geographic proximity of streams. For example, while some exceptions were observed, nearly all *O*. *mykiss* populations (including *O*. *m*. *nelsoni*) clustered separately from most of the SMO populations and *O*. *clarkii*. Trout from the Río Acaponeta, the southern-most population, clustered with trout from Mexican hatcheries within the *O*. *mykiss* lineage in both trees. Trout populations from tributaries of the Río Fuerte did not cluster together, but rather were most similar to populations from the ríos Sinaloa and Conchos and, with marginal bootstrap support, the Río San Lorenzo/Río Piaxtla cluster.

All populations from the ríos Casas Grandes, Yaqui and Mayo, as well as two tributaries of the Río Conchos, were separated by a long, well-supported internal branch that was similar to that separating *O*. *clarkii* from all the other trout species. In addition, strong support was observed for a division between populations from the northern and southern Río Yaqui tributaries (S1 Fig). Populations from ríos Fuerte, Sinaloa and Culiacán (nominal *O*. *chrysogaster*), along with the population from Río Conchos-Arroyo El Molino, formed a cluster in both dendrograms. Also consistent with the PCA, strong support was found for a cluster of populations from the ríos San Lorenzo and Piaxtla.

**Fig 7:**
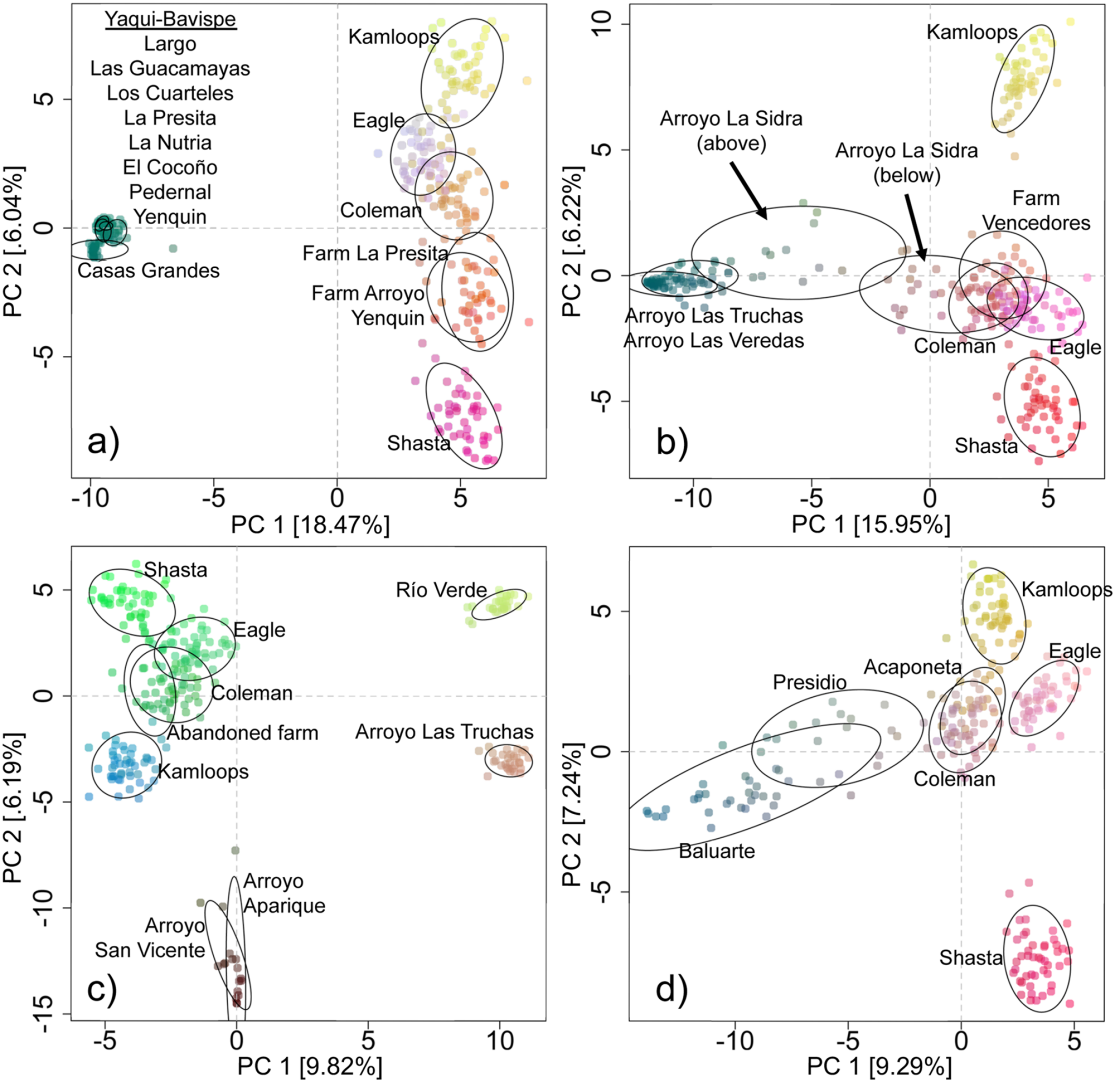
Principal components analysis of allele frequencies. Each plot includes populations from four hatchery rainbow trout stocks raised in California (Coleman, Kamloops, Eagle Lake, and Mount Shasta) and a) all Río Yaqui-Río Bavispe and Río Casas Grandes populations, and samples from “Truchas La Presita” and “Arroyo Yenquin” hatcheries; b) all Río Fuerte populations, samples from an abandoned hatchery located on the Río Fuerte-Arroyo Aparique, and samples from Centro Acuícola Guachochi; c) all Río San Lorenzo populations and samples from “Piscicultura Vencedores” hatchery; d) populations from the three southernmost basins, ríos Presidio, Baluarte, and Acaponeta. The difference in color (red, blue and green) between clusters indicates divergence using the first three principal components.

**Fig 8:**
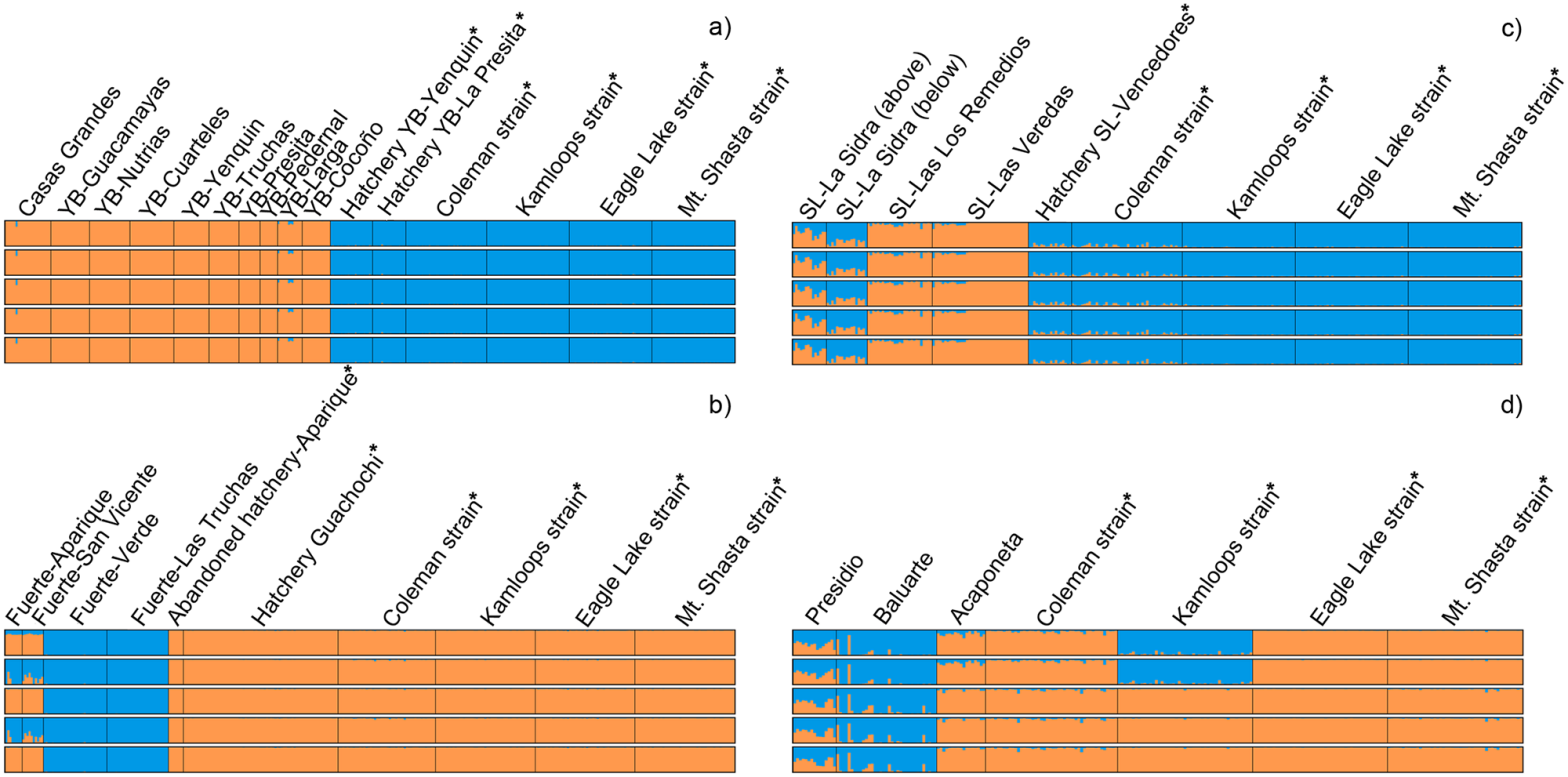
STRUCTURE analysis of hatchery rainbow trout ancestry. Estimated membership fraction (represented by color proportions) from four hatchery rainbow trout stocks raised in California (Coleman, Kamloops, Eagle Lake, and Mount Shasta) and a) all Río Yaqui-Río Bavispe and Río Casas Grandes populations, and samples from “Truchas La Presita” and “Yenquin” hatcheries; b) all Río Fuerte populations, samples from an abandoned hatchery located at the Río Fuerte-Arroyo Aparique, and samples from “Centro Acuícola Guachochi”; c) all Río San Lorenzo populations and samples from “Piscicultura Vencedores” hatchery; d) populations from the three southernmost basins, ríos Presidio, Baluarte, and Acaponeta. Horizontal plots represent unique runs visualized with DISTRUCT. Each thin, colored, vertical line represents one individual. Populations are separated by vertical black lines. Five iterations of each value of *K* (*K* = 2–7) are shown. Hatchery trout populations are indicated with *.

Results from the PCA and STRUCTURE analyses indicated that fish raised at all the Mexican hatcheries sampled for this study, including the nominal *O*. *chrysogaster* from the “Centro Acuícola Guachochi”, corresponded to *O*. *mykiss* and were closely related to hatchery rainbow trout strains commonly raised in the United States (Figs 7 and 8). Introgression from hatchery rainbow trout was present in some native Mexican trout populations (Figs 7 and 8; S2 Table), but was mostly localized in tributaries where rainbow trout hatcheries occur, particularly in the ríos Fuerte and San Lorenzo. The southernmost populations in the ríos Presidio and Acaponeta also had substantial *O*. *mykiss* ancestry indicated by the STRUCTURE analysis, even though no hatchery trout samples were available from these basins, with the Acaponeta population almost pure *O*. *mykiss*.

## Discussion

Here, we again confirm the significant genetic diversity present in trout populations inhabiting northwestern Mexico. Clustering analyses of data from over 100 genetic markers further indicates that there exist at least five major genetic lineages of native trout in Mexico. These clusters originated from at least two, and possibly three, separate colonization events of basins in Northwestern Mexico. The first event gave rise to *O*. *m*. *nelsoni*, the second event to the trout populations of the northern and central SMO. Genetic similarity of the southern-most SMO populations with *O*. *mykiss* could be the result of a third, more recent colonization event by steelhead from California or further north, or could be due to introgression by or naturalization of imported hatchery rainbow trout. We also found significant divergence between native trout from the SMO and populations of the widespread *O*. *mykiss*, and from the other three described species occupying rivers tributary to the Gulf of California (*O*. *apache*, *O*. *gilae*, and *O*. *clarkii*).

The great diversity in the Mexican trout complex has been the subject of a number of previous investigations. Substantial questions have remained, however, about the relationships among Mexican trout populations [7–9,13,16], and between them and other trout species [10,17,18,66]. Only two trout taxa from Mexico have been formally described—Nelson’s trout, *O*. *mykiss nelsoni* [3] and the Mexican golden trout, *O*. *chrysogaster* [4]—and both are protected by Mexican law [5]. Nelson’s trout is native to the Río Santo Domingo in the Sierra de San Pedro Mártir in northern Baja California [3,16,67], but its taxonomic status as a subspecies has been questioned [68]. The STRUCTURE, PCA and dendrogram analyses all indicate that Nelson’s trout is, in fact, a population of *O*. *mykiss* and not closely related to SMO trout or the other species. This is in agreement with earlier work that originally described Nelson’s trout as a new species (*Salmo nelsoni*) [3], but later concluded that they were closely related to *Salmo irideus* [66], now referred to *O*. *mykiss irideus* [8,69]. Moreover, the dendrograms indicated a stronger similarity of *O*. *m*. *nelsoni* to steelhead DPS populations from southern California than to any other populations of steelhead or hatchery rainbow trout. This observation strongly suggests that trout in Baja California are the result of a radiation of coastal steelhead through southern California and at least as far south as the Sierra de San Pedro Mártir in Baja California, and not the result of an earlier radiation that gave rise to the trout populations in tributaries of the Gulf of California. Even though there was not strong genetic differentiation of *O*. *m*. *nelsoni* from other *O*. *mykiss* with the markers used here, it is important to note that it had the lowest genetic diversity and the highest *F*_*ST*_ values of all *O*. *mykiss* populations studied (S2 and S3 Tables), an indication of small effective population size (*N*_*E*_) and a long period of isolation.

Extremely strong genetic differentiation among Mexican trout populations from the SMO was also observed, not only between basins but also among localities/tributaries within basins. Higher pairwise *F*_*ST*_ estimates were observed between SMO trout populations (mean *F*_*ST*_ = 0.351–0.684) than between *O*. *mykiss* populations (mean *F*_*ST*_ = 0.244–0.527), which is likely due primarily to small effective population sizes of SMO trout and consequent strong genetic drift, which is consistent with the lower diversity found at both microsatellite and SNP markers. However, polymorphisms, SNPs in particular, are frequently not maintained even in closely related species [50,70], so it is likely that at least some of the lower genetic diversity is the result of ascertainment bias due to the discovery of the SNP markers, and some of the microsatellites, in *O*. *mykiss*.

Within the SMO trout, we found at least four well-differentiated genetic clusters. Trout from the ríos Yaqui, Mayo and Casas Grandes, and the northern Río Conchos tributaries form a genetic cluster that is very divergent from other SMO trout populations and the other trout species, which is concordant with previous work on diversity in Mexican trout using morphological variation [8], mtDNA [13,17] and microsatellites [10,18]. Our analysis also confirmed the previously observed local structure within the Río Yaqui [10,13,18,71], with genetic groups defined by the two main tributaries: populations from the Río Bavispe to the north and those from the Río Sirupa to the south (S1 Fig). Trout from the Río Casas Grandes seem to be closely related to trout from Río Bavispe, while trout from the Río Mayo are allied with populations from the Río Sirupa. These relationships between the Río Bavispe/Río Casas Grandes and the Río Sirupa/Río Mayo were also described by Nielsen and Sage [10], who hypothesized that they may be due to multiple, natural environmental events, such as stream capture, that interconnected tributaries of the ríos Yaqui, Casas Grandes, Mayo, and Conchos basins [71], permitting movement of multiple fish species between drainages [72,73]. However, neither their nor the present results can rule out anthropogenic inter-basin transplants [12] as an explanation and both factors could have played a role in creating the observed patterns.

Populations from ríos Fuerte, Sinaloa and Culiacán, identified as *O*. *chrysogaster*, formed a single group, with the exception of trout from two tributaries of the Río Fuerte (arroyos San Vicente and Aparique), which were somewhat distinct in all analyses and appear more similar to *O*. *mykiss* or populations from the ríos San Lorenzo and Piaxtla. This is likely the result of genetic introgression from hatchery rainbow trout, but could also be due, at least partly, to reduced genetic variation or retention of ancestral polymorphisms. However, there was no evidence of grouping by basin for *O*. *chrysogaster* populations, as found by Ruiz-Campos *et al*. [8] and Camarena-Rosales *et al*. [13], but rather a strong association between geographically adjacent tributaries of different basins (PCA not shown, S2 Fig). This pattern may be the result of one of a combination of the above listed explanations, but resolving them requires more detailed investigation.

Trout from the Río Conchos were originally described as “cutthroat type” [74], but then were not seen for decades, before they were rediscovered recently after exhaustive survey efforts [9]. Genetic analyses did not indicate that populations from this basin are related to cutthroat trout, but that they are more closely related to other SMO trout. The upper Río Conchos is one of only a few sites on the eastern side of the Continental Divide of North America, and the only one in Mexico, that harbors fish from the Pacific salmon and trout genus *Oncorhynchus*. Trout from the Río Conchos tributaries Rituchi and Ureyna grouped tightly with Río Yaqui-Río Bavispe trout, which are in close proximity (S1 Fig). In contrast, fish from the southern Río Conchos tributary El Molino clustered closely with trout from the Río Verde, a tributary of the Río Fuerte, and were identified as part of the *O*. *chrysogaster* lineage with multiple analyses. These two tributaries are geographically adjacent (S2 Fig) on opposite sides of the Continental Divide and stream capture events seem a likely explanation for this genetic similarity. Such movement between headwater streams of the ríos Fuerte and Conchos has been previously reported in other freshwater fish species [72,73,75].

Trout populations from the ríos San Lorenzo and Piaxtla formed another strongly supported cluster in all analyses. However, trout from Arroyo La Sidra in the Río San Lorenzo were heavily introgressed by hatchery rainbow trout (Figs 7 and 8), and fish below the hatchery (downstream) were more introgressed than those upstream. This is consistent with a previous report that estimated a considerable number of migrants (*Nm* = 2.7) between native and exotic trout in this stream [18]. No introgression by hatchery trout was detected in other Río San Lorenzo populations, indicating that exotic trout have not extended their range much beyond the immediate vicinity of the hatchery.

Trout from the ríos Presidio, Baluarte and Acaponeta were more similar to *O*. *mykiss* than to the other SMO lineages. Hybridization between trout from these southern drainages and exotic rainbow trout has been previously reported [18,30]. The genetic results indicate that trout from the ríos Presidio and Baluarte have hatchery ancestry, although the dendrogram (Fig 6), PCA (Fig 7) and STRUCTURE analyses (Fig 8) suggest that the Río Presidio population in particular may be at least partially of native ancestry. While these patterns are most likely the result of hybridization between native and hatchery rainbow trout, they could also be due to a natural colonization event by anadromous *O*. *mykiss* from the Pacific coast that was more recent than that which gave rise to the other SMO trout. The ríos Presidio and Baluarte are at the extreme southern edge of the known range of trout in Mexico and they are also likely the first perennial streams that anadromous steelhead venturing south from their current contiguous range would encounter. Distinguishing natural colonization from introgression by introduced hatchery trout will require more detailed analysis.

Trout from Río Acaponeta clustered with *O*. *mykiss* in every analysis and, more specifically, with hatchery rainbow trout strains. This provides strong evidence that Río Acaponeta trout are descended directly from hatchery rainbow trout or recent migrants as described above, and that if there was formerly a native population related to the other SMO trout, it is completely introgressed.

The Centro Acuícola Guachochi hatchery facility in the state of Chihuahua was originally dedicated to the production of hatchery rainbow trout but, in 2006, started a program to breed and raise *O*. *chrysogaster* for conservation purposes [76]. Unfortunately, genetic results indicated that trout from this program do not correspond to *O*. *chrysogaster* or any other native SMO lineages. In contrast, they clustered tightly with hatchery rainbow trout, indicating collection from a stream with previously unrecognized hatchery trout stocking and consequent incorrect identification, or introgression from exotic rainbow trout also raised at the hatchery.

The first documented introduction of non-native trout into Mexican waters was in 1886, when ~33,000 eggs were imported from the Baird Station hatchery on the McCloud River, California, United States [77]. The total amount of rainbow trout aquaculture production in Mexico is unknown, but reports indicate that there are ~40 facilities that produce 110 tons of fish a year in the state of Durango alone and ~82 facilities that produce 184 tons a year in the state of Chihuahua [78,79]. Both of these states also possess native trout populations. Introgression from non-native rainbow trout was documented in most of the tributaries in the study area with established hatcheries, but at variable levels and limited geographic distance from the hatcheries. Nevertheless, genetic integrity of native trout from northwestern Mexico was still maintained in most watersheds. The patterns of genetic relationships between populations of Mexican trout documented here will be crucial to guide effective conservation strategies for these fishes.

In summary, we present the first comprehensive genetic analysis of trout from northwestern Mexico. We used data from over 100 markers, a combination of SNPs and microsatellites, to clearly delineate at least five distinct species-level genetic lineages in Mexico, corresponding to the two named taxa, Mexican Golden and Nelson’s Trout, and at least three unnamed taxa in the Sierra Madre Occidental region. We further clarified the taxonomic status of trout from northern Baja California, unambiguously identifying them as the southern-most population of the coastal California steelhead lineage. In addition, we identify the taxonomic affiliation of fish from the Atlantic Ocean-draining Rio Conchos basin, finding that they are of mixed origin, with one representing a previously unknown population of, at least partial, Mexican Golden Trout ancestry. The data further allowed us to evaluate the ancestry of trout populations that could have been affected by introgression by imported hatchery rainbow trout. This analysis found that, while there was some evidence of introgression of native trout by these hatchery imports, it was generally restricted to populations in the local vicinity of the hatcheries. However, several populations at the southern-most extent of the range were dominated by *O*. *mykiss* ancestry, although it is possible that this is due to a more recent anadromous colonization event.

## Supporting Information

S1 Fig Sample locations in the Northern Sierra Madre Occidental (NSMO). Five watersheds are indicated: Río Casas Grandes (light green), Río Yaqui (dark green), Río Mayo (olive green), Río Fuerte (blue), and Río Conchos (red).
(TIFF)

S2 Fig Sample locations of *Oncorhynchus chrysogaster* and Río Conchos trout. Four watersheds are indicated: Ríos Fuerte, Sinaloa and Culiacán (blue), and Río Conchos (red).
(TIFF)

S1 Table Populations in this study from north to south. *N*: total number of samples; Lat: Latitude; Long: Longitude; *: estimated location; DPS: Distinct population segment.
(XLSX)

S2 Table Summary statistics. *N*: number of individuals successfully genotyped with the microsatellite panel and the SNP panel respectively; Expected (*H*_*E*_) and observed (*H*_*O*_) heterozygosity; allelic richness (*A*_*R*_); percentage of polymorphic loci (*P*) at frequencies 0.95 and 0.99.
(XLSX)

S3 Table Pairwise *F*_*ST*_ estimates for all populations and hatchery strains (below diagonal) and significance of *p*-values (above diagonal). Lines indicate breaks for the main groups (*O*. *mykiss*, *O*. *m*. *nelsoni*, NSMO, *O*. *chrysogaster*, SSMO, *O*. *apache*, *O*. *gilae*, and *O*. *clarkii* ssp., Mexican hatcheries, and California hatcheries.
(XLSX)

S4 Table Genotyping assays used in this study. Original reference is reported. *F*_*ST*_ by locus using Weir & Cockerham’s (1984) estimator. WSU: Washington State University; CRITFC: Columbia River Inter-Tribal Fish Commission.
(XLSX)

## References

[1] Mayden RL, Wood RM (1995) Systematics, species concepts, and the evolutionarily significant unit in biodiversity and conservation biology In: Nielsen JL, editor. Evolution and the aquatic ecosystem: Defining unique units in population conservation. American Fisheries Society Symposium 17. Bethesda, Maryland: American Fisheries Society pp. 58–113.

[2] Behnke RJ. Trout and salmon of North America. 1st ed New York: Chanticleer ress; 2002.

[3] Evermann BW (1908) Descriptions of a new species of trout (Salmo nelsoni) and a new Cyprinodont (Fundulus meeki) with notes on other fishes from lower California. Proc Biol Soc Washingt 21: 19–30.

[4] Needham PR, Gard R (1964) A new trout from Central Mexico: *Salmo chrysogaster*, the Mexican golden trout. Copeia 1964: 169–173.

[5] SEMARNAT (2000) Norma oficial mexicana NOM- 059-ECOL- 2001, protección ambiental- especies nativas de México de flora y fauna silvestres- Categorías de riesgo y especificaciones para su inclusión, exclusión o cambio- Lista de especies en riego.

[6] IUCN (2010) IUCN Red List of Threatened Species. Version 2010.4. www.iucnredlist.org. Downloaded on 21 February 2011.

[7] Hendrickson DA, Espinosa-Peérez H, Findley LT, Forbes W, Tomelleri JR, Mayden RL, et al (2002) Mexican native trouts: a review of their history and current systematic and conservation status. Rev Fish Biol Fish 12: 273–316.

[8] Ruiz-Campos G, Camarena-Rosales F, Varela-Romero A, Sánchez-Gonzales S, De la Rosa-Vélez J (2003) Morphometric variation of wild trout populations from northwestern Mexico (Pisces: Salmonidae). Rev Fish Biol Fish 13: 91–110.

[9] Hendrickson DA, Neely DA, Mayden RL, Anderson K, Brooks JE, Camarena-Rosales F, et al (2006) Conservation of Mexican native trout and the discovery, status, protection and recovery of the Conchos trout, the first native Oncorhynchus of the Atlantic drainage in Mexico. In: Lozano-Vilano M de L, Contreras-Balderas AJ, editors. Universidad Autonoma de Nuevo Leon, Mexico pp. 162–201.

[10] Nielsen JL, Sage GK (2001) Microsatellite analyses of the trout of northwest Mexico. Genetica 111: 269–278. 11841172 10.1023/a:1013777701213

[11] Cavender TM, Miller RR (1982) Salmo australis, a new species of fossil salmonid from southwestern Mexico In: Smith GR, editor. Contributions from the Museum of Paleontology, The University of Michigan, Vol. 26 pp. 1–17.

[12] Behnke RJ (1992) Native trout of western North America. Bethesda: American Fisheries Society Monograph 6.

[13] Camarena-Rosales F, Ruiz-Campos G, De La Rosa-Vélez J, Mayden RL, Hendrickson DA, Varela-Romero A, et al (2007) Mitochondrial haplotype variation in wild trout populations (Teleostei: Salmonidae) from northwestern Mexico. Rev Fish Biol Fish 18: 33–45.

[14] Phillips R, Rab P (2001) Chromosome evolution in the Salmonidae (Pisces): an update. Biol Rev 76: 1–25. 11325050 10.1017/s1464793100005613

[15] Loudenslager EJ, Rinne JN, Gall GAE, David RE (1986) Biochemical genetic studies of native Arizona and New Mexico trout. Southwest Nat 31: 221–234.

[16] Ruiz-Campos G, Pister EP (1995) Distribution, habitat, and current status of the San Pedro Martir rainbow trout, Oncorhynchus mykiss nelsoni (Evermann). Bull South Calif Acad Sci 94: 131–148.

[17] Nielsen JL, Fountain MC, Favela JC, Cobble K, Jensen BL (1998) *Oncorhynchus* at the southern extent of their range: a study of mtDNA control-region sequence with special reference to an undescribed subspecies of *O*. *mykiss* from Mexico. Environ Biol Fishes 51: 7–23.

[18] De los Santos-Camarillo AB (2008) Definición de unidades taxonómicas en el complejo de truchas del noroeste de México, mediante el análisis de marcadores microsatálites. Masters thesis, Centro de Investigaciones Biológicas del Noroeste, S.C., La Paz, Baja California Sur, Mexico.

[19] FAO (2005–2014) Cultured Aquatic Species Information Programme. Oncorhynchus mykiss. Text by Cowx, I. G. In: FAO Fisheries and Aquaculture Department [online]. Rome. Updated 15 June 2005. [Cited 5 June 2014]. Available: http://www.fao.org/fishery/culturedspecies/Oncorhynchus_mykiss/en

[20] Rhymer JM, Simberloff D (1996) Extinction by hybridization and introgression. Annu Rev Ecol Syst 27: 83–109.

[21] USFWS (2003) Gila trout recovery plan. 3rd revision Albuquerque, New Mexico.

[22] Garza JC, Pearse DE (2008) Population genetic structure of Oncorhynchus mykiss in the California Central Valley. University of California, Santa Cruz and NOAA, Southwest Fisheries Science Center Available at https://swfsc.noaa.gov/publications/FED/00993.pdf

[23] Clemento AJ, Anderson EC, Boughton D, Girman DJ, Garza JC (2009) Population genetic structure and ancestry of *Oncorhynchus mykiss* populations above and below dams in south-central California. Conserv Genet 10: 1321–1336.

[24] Allendorf FW, Leary RF, Hitt NP, Knudsen KL, Lundquist LL, Spruell P (2004) Intercrosses and the U.S. Endangered Species Act: should hybridized populations be included as westslope cutthroat trout? Conserv Biol 18: 1203–1213.

[25] Leary RF, Allendorf FW, Knudsen KL (1985) Developmental instability and high meristic counts in interspecific hybrids of salmonid fishes. Evolution 39: 1318–1326. 28564256 10.1111/j.1558-5646.1985.tb05697.x

[26] Rubidge E, Corbett P, Taylor EB (2001) A molecular analysis of hybridization between native westslope cutthroat trout and introduced rainbow trout in southeastern British Columbia, Canada. J Fish Biol 59(Suppl. A): 42–54.

[27] Weigel DE, Peterson JT, Spruell P (2003) Introgressive hybridization between native cutthroat trout and introduced rainbow trout. Ecol Appl 13: 38–50.

[28] Shepard BB, May BE, Urie W (2005) Status and conservation of westslope cutthroat trout within the western United States. North Am J Fish Manag 25: 1426–1440.

[29] Dowling TE, Childs MR (1992) Impact of hybridization on a threatened trout of the southwestern United States. Conserv Biol 6: 355–364.

[30] Escalante MA, García-De-León FJ, Dillman CB, de los Santos-Camarillo A, George A, Barriga-Sosa I de los A, et al (2014) Genetic introgression of cultured rainbow trout in the Mexican native trout complex. Conserv Genet 15: 1063–1071.

[31] Busby PJ, Wainwright TC, Bryant GJ, Lierheimer LJ, Waples RS, Waknitz FW, et al (1996) Status review of West Coast steelhead from Washington, Idaho, Oregon, and California NOAA Technical Memorandum NOAA-TM-NMFS-NWFSC-27. US Department of Commerce.

[32] Bjorkstedt EP, Spence BC, Garza JC, Hankin DG, Fuller D, Jones WE, et al (2005) An analysis of historical population structure for evolutionarily significant units of Chinook salmon, coho salmon, and steelhead in the north-central California coast recovery domain NOAA Technical Memorandum NOAA-TM-NMFS-SWFSC-382. US Department of Commerce.

[33] Wilson WD, Turner TF (2009) Phylogenetic analysis of the Pacific cutthroat trout (Oncorhynchus clarki ssp.: Salmonidae) based on partial mtDNA ND4 sequences: a closer look at the highly fragmented inland species. Mol Phylogenet Evol 52: 406–415. 10.1016/j.ympev.2009.03.018 19341807

[34] Aguilar A, Garza JC (2006) A comparison of variability and population structure for major histocompatibility complex and microsatellite loci in California coastal steelhead (Oncorhynchus mykiss Walbaum). Mol Ecol 15: 923–937. 16599957 10.1111/j.1365-294X.2006.02843.x

[35] Garza JC, Gilbert-Horvath EA, Spence BC, Williams TH, Fish H, Gough SA, et al (2014) Population structure of steelhead in coastal California. Trans Am Fish Soc 143: 134–152.

[36] McConnell SK, O'Reilly P, Hamilton L, Wright JM, Bentzen P (1997) Polymorphic microsatellite loci from Atlantic salmon (Salmo salar): genetic differentiation of North American and European populations. Can J Fish Aquat Sci 52: 1863–1872.

[37] McConnell SK, Hamilton LC, Morris DB, Cook D, Paquet D, Bentzen P, et al (1995) Isolation of salmonid microsatellite loci and their application to the population genetics of Canadian east coast stocks of Atlantic salmon. Aquaculture 137: 19–30.

[38] Morris DB, Richard KR, Wright JM (1996) Microsatellites from rainbow trout (Oncorhynchus mykiss) and their use for genetic study of salmonids. Can J Fish Aquat Sci 53: 120–126.

[39] O’Reilly PT, Hamilton LC, McConnell SK, Wright JM (1996) Rapid analysis of genetic variation in Atlantic salmon (Salmo salar) by PCR multiplexing of dinucleotide and tetranucleotide microsatellites. Can J Fish Aquat Sci 53: 2292–2298.

[40] Scribner KT, Gust JR, Fields RL (1996) Isolation and characterization of novel salmon microsatellite loci: cross-species amplification and population genetic applications. Can J Fish Aquat Sci 53: 833–841.

[41] Condrey MJ, Bentzen P (1998) Characterization of coastal cutthroat trout (Oncorhynchus clarki clarki) microsatellites and their conservation in other salmonids. Mol Ecol 7: 787–789. 9640655

[42] Small MP, Beacham TD, Withler RE, Nelson RJ (1998) Discriminating coho salmon (Oncorhynchus kisutch) populations within the Fraser River, British Columbia, using microsatellite DNA markers. Mol Ecol 7: 141–155.

[43] Smith CT, Koop BF, Nelson RJ (1998) Isolation and characterization of coho salmon (Oncorhynchus kisutch) microsatellites and their use in other salmonids. Mol Ecol 7: 1614–1616. 9819912

[44] Banks MA, Blouin MS, Baldwin BA, Rashbrook VK, Fitzgerald HA, Blankenship SM, et al (1999) Isolation and inheritance of novel microsatellites in Chinook salmon (Oncorhynchus tschawytscha). J Hered 90: 281–288.

[45] Williamson KS, Cordes JF, May B (2002) Characterization of microsatellite loci in Chinook salmon (Oncorhynchus tshawytscha) and cross-species amplification in other salmonids. Mol Ecol Notes 2: 17–19.

[46] Pearse DE, Donohoe CJ, Garza JC (2007) Population genetics of steelhead (Oncorhynchus mykiss) in the Klamath River. Environ Biol Fishes 80: 377–387.

[47] Wenburg JK, Bentzen P, Foote CJ (1998) Microsatellite analysis of genetic population structure in an endangered salmonid: the coastal cutthroat trout (Oncorhynchus clarki clarki). Mol Ecol 7: 733–749. 9640655

[48] Nielsen JL, Sage GK (2002) Population genetic structure in Lahontan cutthroat trout. Trans Am Fish Soc 131: 376–388.

[49] Aguilar A, Garza JC (2008) Isolation of 15 single nucleotide polymorphisms from coastal steelhead, Oncorhynchus mykiss (Salmonidae). Mol Ecol Resour 8: 659–662. 10.1111/j.1471-8286.2007.02038.x 21585863

[50] Campbell NR, Overturf K, Narum SR (2009) Characterization of 22 novel single nucleotide polymorphism markers in steelhead and rainbow trout. Mol Ecol Resour 9: 318–322. 10.1111/j.1755-0998.2008.02376.x 21564638

[51] Abadía-Cardoso A, Clemento AJ, Garza JC (2011) Discovery and characterization of single-nucleotide polymorphisms in steelhead/rainbow trout, *Oncorhynchus mykiss* . Mol Ecol Resour 11(Suppl. 1): 31–49. 10.1111/j.1755-0998.2010.02971.x 21429161

[52] Nei M (1978) Estimation of average heterozygosity and genetic distance from a small number of individuals. Genetics 89: 583–590. 17248844 10.1093/genetics/89.3.583 PMC1213855

[53] Rousset F (2008) GENEPOP ‘007: a complete re-implementation of the GENEPOP software for Windows and Linux. Mol Ecol Resour 8: 103–106. 10.1111/j.1471-8286.2007.01931.x 21585727

[54] Belkhir K, Borsa P, Chikhi L, Raufaste N, Bonhomme F (2004) GENETIX 4.05, logiciel sous Windows™ pour la génétique des populations Laboratoire Génome, Populations, Interactions, CNRS UMR 5000, Université de Montpellier II, Montpellier (France).

[55] Goudet J (2005) Hierfstat, a package for R to compute and test hierarchical F-statistics. Mol Ecol Notes 5: 184–186.

[56] R Development Core Team (2011) A Language and Environment for Statistical Computing. Available: http://www.r-project.org.

[57] Pritchard JK, Stephens M, Donnelly P (2000) Inference of population structure using multilocus genotype data. Genetics 155: 945–959. 10835412 10.1093/genetics/155.2.945 PMC1461096

[58] Jakobsson M, Rosenberg NA (2007) CLUMPP: a cluster matching and permutation program for dealing with label switching and multimodality in analysis of population structure. Bioinformatics 23: 1801–1806. 17485429 10.1093/bioinformatics/btm233

[59] Rosenberg NA (2004) DISTRUCT: a program for the graphical display of population structure. Mol Ecol Notes 4: 137–138.

[60] Excoffier L, Lischer HEL (2010) Arlequin suite ver 3.5: a new series of programs to perform population genetics analyses under Linux and Windows. Mol Ecol Resour 10: 564–567. 10.1111/j.1755-0998.2010.02847.x 21565059

[61] Jombart T (2008) adegenet: a R package for the multivariate analysis of genetic markers. Bioinformatics 24: 1403–1405. 10.1093/bioinformatics/btn129 18397895

[62] Cailliez F, Pagès JP (1976) Introduction à l’Analyse des Donnèes. SMASH, Paris.

[63] Felsenstein J (2005) PHYLIP (Phylogeny Inference Package) version 3.6. Available: http://evolution.genetics.washington.edu/phylip.html

[64] Cavalli-Sforza LL, Edwards AWF (1967) Phylogenetic analysis models and estimation procedures. Evolution 21: 550–570. 28563688 10.1111/j.1558-5646.1967.tb03411.x

[65] Hedrick PW (1999) Perspective: highly variable loci and their interpretation in evolution and conservation. Evolution 53: 313–318. 28565409 10.1111/j.1558-5646.1999.tb03767.x

[66] Mayden RL, Dillman CB, Espinosa-Peérez H, Tomelleri JR, Kuhajda BR, Hendrickson DA, et al (2010) Evolution and diversity of trout species in the Sierra Madre Occidental of Mexico In: Carline RF, LoSapio C, editors. Conserving Wild Trout. Proceedings of the Wild Trout X symposium. Bozeman, Montana pp. 134–144.

[67] Snyder JO (1926) The trout of the Sierra San Pedro Martir, Lower California. Univ Calif Publ Zool 21: 419–426.

[68] Miller RR, Minckley WL, Norris SM (2005) Freshwater Fishes of Mexico. Chicago and London: The University of Chicago Press.

[69] Needham PR (1938) Notes on the introduction of *Salmo nelsoni* Evermann into California from Mexico. Trans Am Fish Soc 67: 139–146.

[70] Campbell NR, Amish SJ, Pritchard VL, McKelvey KS, Young MK, Schwartz MK, et al (2012) Development and evaluation of 200 novel SNP assays for population genetic studies of westslope cutthroat trout and genetic identification of related taxa. Mol Ecol Resour 12: 942–949. 10.1111/j.1755-0998.2012.03161.x 22697369

[71] Hendrickson DA, Minckley WL, Miller RR, Siebart DJ, Minckley PH (1980) Fishes of the Río Yaqui Basin, México and United States. J Arizona-Nevada Acad Sci 15: 65–106.

[72] Domínguez-Domínguez O, Vila M, Pérez-Rodríguez R, Remón N, Doadrio I (2011) Complex evolutionary history of the Mexican stoneroller Campostoma ornatum Girard, 1856 (Actinopterygii: Cyprinidae). BMC Evol Biol 11: 153 10.1186/1471-2148-11-153 21639931 PMC3141424

[73] Schönhuth S, Blum MJ, Lozano-Vilano L, Neely DA, Varela-Romero A, Espinosa H, et al (2011) Inter-basin exchange and repeated headwater capture across the Sierra Madre Occidental inferred from the phylogeography of Mexican stonerollers. J Biogeogr 38: 1406–1421.

[74] Cope ED (1886) The most southern salmon. Am Nat 20: 735.

[75] Schönhuth S, Perdices A, Lozano-Vilano L, García-de-León FJ, Espinosa H, Mayden RL. (2014) Phylogenetic relationships of North American western chubs of the genus Gila (Cyprinidae, Teleostei), with emphasis on southern species. Mol Phylogenet Evol 70: 210–230. 10.1016/j.ympev.2013.09.021 24096056

[76] Barriga-Sosa I de los A, Arredondo-Figueroa JL, De-la-Mora GI, Banda-Cortés M, Rendón Gutierrez L, García-de-León FJ. (n.d.) Primeras experiencias del cultivo de la trucha dorada (Oncorhynchus chrysogaster) Retrieved from http://www.izt.uam.mx/pexpa/htms/proyect3.htm.

[77] Arredondo-Figueroa J (1983) Especies animales acuáticas de importancia nutricional, introducidas en México. Biotica 9: 23–39.

[78] Diaz L. Compra Chihuahua el 70% del pescado que consume. El Diario de Chihuahua. 2010. Available: http://www.eldiariodechihuahua.mx

[79] México: Gobierno de Durango impulsa la producción y el consumo de la trucha arco-iris. Aquahoy. 2010. Available: http://www.durango.gob.mx/es/publicaciones/Gobierno_del_Estado_impulsa_la_produccion_y_el_consumo_de_la_trucha_arco_iris

