# Supplementary figures and images for: Genetic Structure of Pacific Trout at the Extreme Southern End of Their Native Range
Source: PLoS One. 2015 Oct 28;10(10):e0141775. doi: 10.1371/journal.pone.0141775 (PMC4624944; doi:10.1371/journal.pone.0141775)

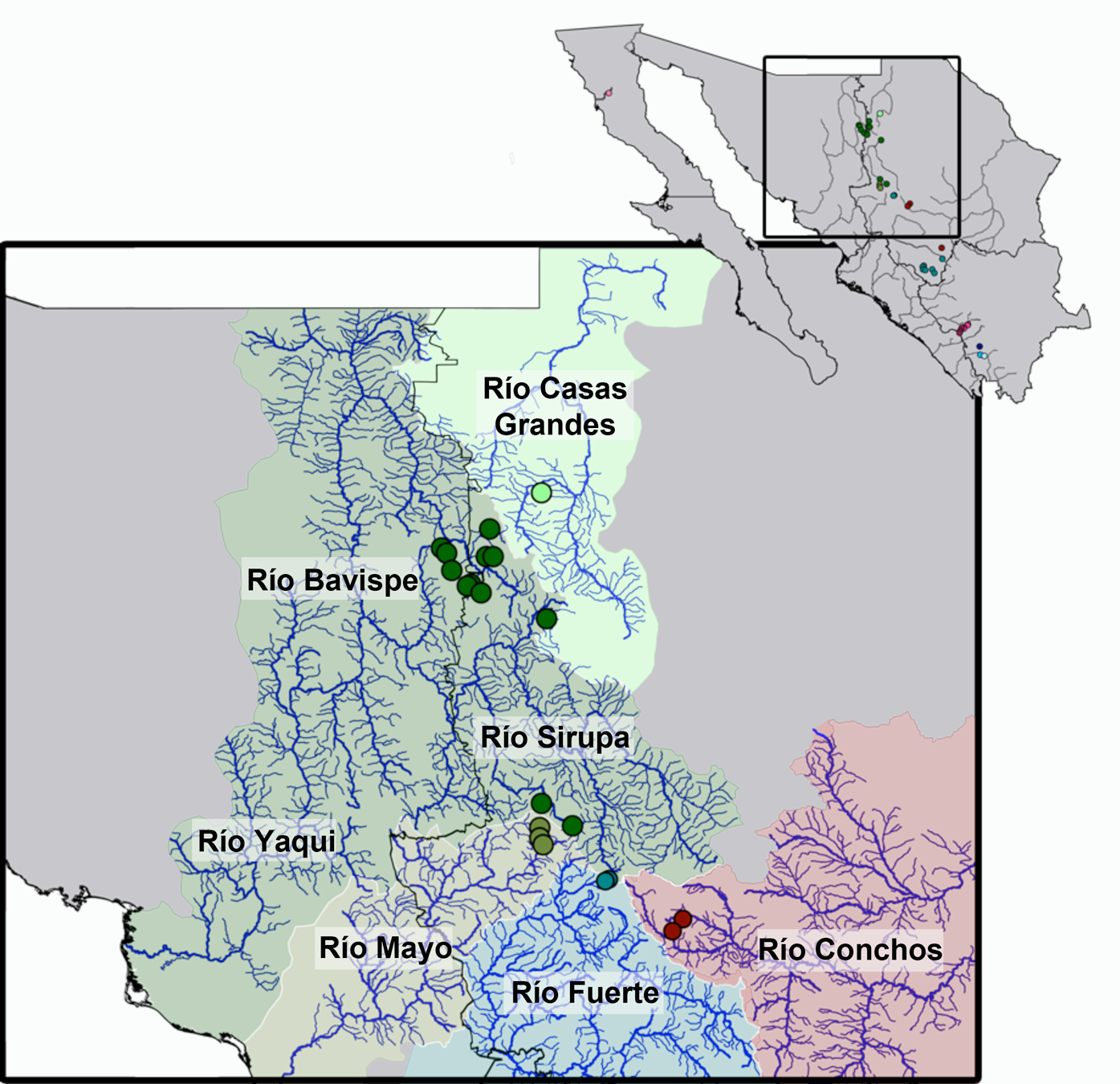

Supplement: S1 Fig — Five watersheds are indicated: Río Casas Grandes (light green), Río Yaqui (dark green), Río Mayo (olive green), Río Fuerte (blue), and Río Conchos (red). (TIFF) [file pone.0141775.s001.tiff]

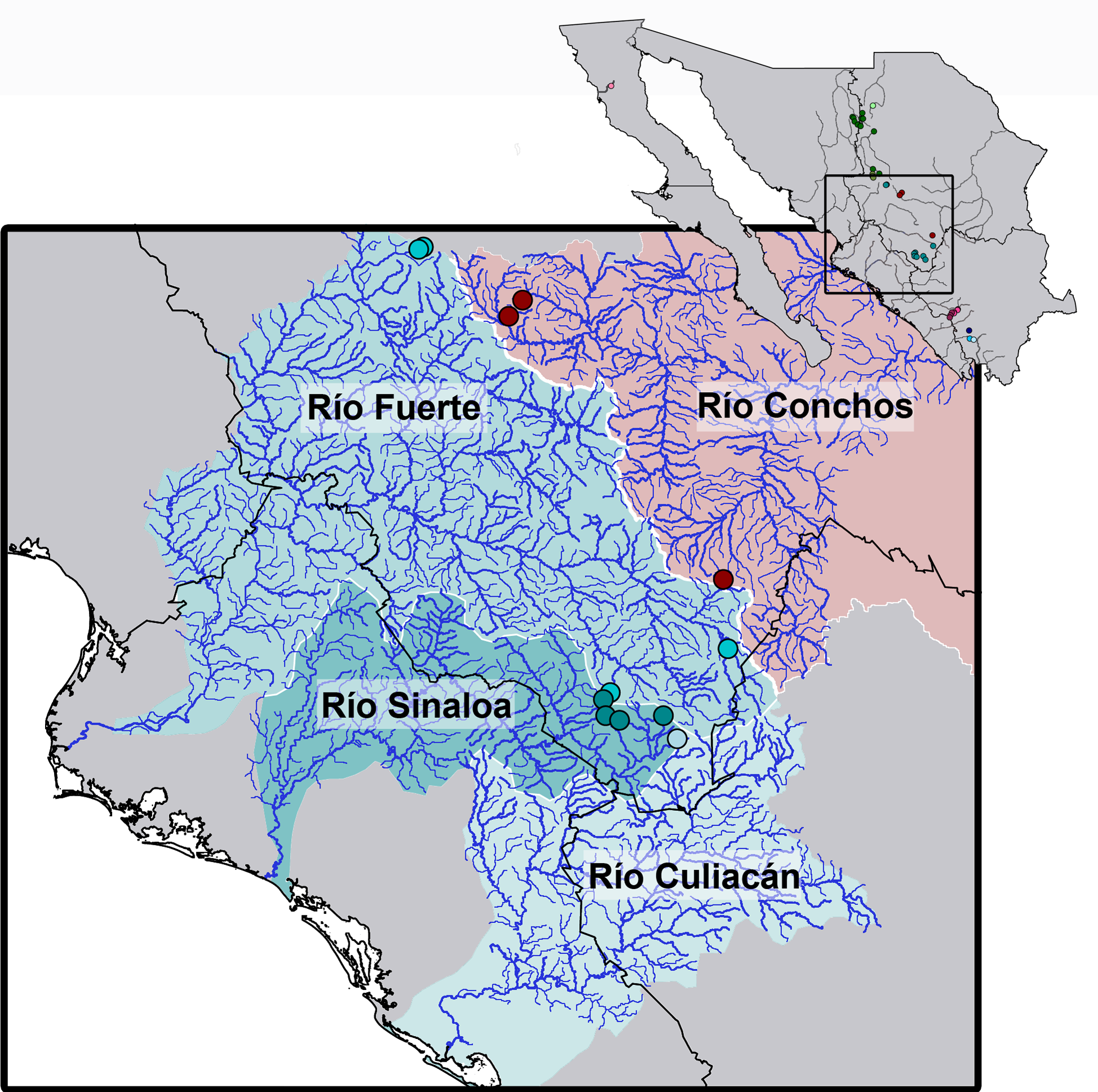

Supplement: S2 Fig — Four watersheds are indicated: Ríos Fuerte, Sinaloa and Culiacán (blue), and Río Conchos (red). (TIFF) [file pone.0141775.s002.tiff]
